# Immunogenicity of Inactivated SARS-CoV-2 Vaccines in Patients With Rheumatoid Arthritis: A Case Series

**DOI:** 10.3389/fpubh.2022.875558

**Published:** 2022-04-25

**Authors:** Ting Zhao, Jiayan Shen, Youyang Zhu, Xiaofang Tian, Guangfen Wen, Yuanyuan Wei, Bonan Xu, Chenyang Fu, Zhaohu Xie, Yujiang Xi, Zhenmin Li, Jiangyun Peng, Yang Wu, Xiaohu Tang, Chunping Wan, Lei Pan, Zhaofu Li, Dongdong Qin

**Affiliations:** ^1^School of Basic Medical Sciences, Yunnan University of Chinese Medicine, Kunming, China; ^2^The First School of Clinical Medicine, Yunnan University of Chinese Medicine, Kunming, China; ^3^The Third Affiliated Hospital, Yunnan University of Chinese Medicine, Kunming, China; ^4^The Second School of Clinical Medicine, Yunnan University of Chinese Medicine, Kunming, China

**Keywords:** COVID-19, inactivated SARS-CoV-2 vaccines, rheumatoid arthritis, neutralizing antibodies, immunogenicity

## Abstract

**Objectives:**

Attenuated humoral response to mRNA SARS-CoV-2 vaccines has been reported in some patients with autoimmune disease, e.g., rheumatoid arthritis (RA). However, data of immune responses to inactivated SARS-CoV-2 vaccine in the RA population are still unknown. Herein, the safety and immunogenicity of inactivated SARS-CoV-2 vaccines in RA patients were analyzed.

**Methods:**

Seventy five RA patients and 26 healthy controls (HC) were respectively recruited from Yunnan Provincial Hospital of Traditional Chinese Medicine and the community in Kunming city. Neutralizing Antibody (NAb) Test ELISA kit was used to measure the percentage of inhibition. AKA (anti-keratin antibody) positivity was detected using indirect immunofluorescence. Rheumatoid factor (RF)-IgA was detected by ELISA. RF-IgG, RF-IgM, and anti-cyclic citrullinated peptide (CCP) antibodies were measured by chemiluminescence. ESR (erythrocyte sedimentation rate) was detected by ESR analyzer. C-RP (c-reactive protein) was detected by immunoturbidimetry. NEUT% (percentage of neutrophils) and LYMPH% (percentage of percentage) were calculated by a calculation method.

**Results:**

Compared with the HC group, the percentage of inhibition was significantly lower in RA patients receiving two doses of vaccines. Vaccines-induced percentage of inhibition was the lowest in RA patients who had not been vaccinated. In total 80.77% of the HC group had a percentage of inhibition ≧20%, compared with 45.24% of vaccinated RA patients and 6.06% of unvaccinated RA patients. Spearman correlation analysis revealed that antibody responses to SARS-CoV-2 did not differ between RA patients according to their age and disease duration. Furthermore, the results showed that no correlation was found between the percentage of inhibition and indices for RA, including RF-IgA, IgG, IgM; anti-CCP antibody; ESR; C-RP; NEUT% and LYMPH%.

**Conclusion:**

Our study showed inactivated vaccine-induced SARS-COV-2 antibody responses differ in RA patients and healthy subjects, emphasizing the importance of a third or fourth vaccination in RA patients.

## Introduction

Coronavirus disease 2019 (COVID-19) has progressed to a worldwide pandemic and posed enormous challenges to healthcare (1). Since January 2020, the virus has spread rapidly to large parts of China and other countries, which soon captured global attention with the pathogen identified as SARS-CoV-2. According to new data released by WHO (World Health Organization) on 18 March 2022, there have been 464,809,377 confirmed cases, and approximately 6,062,536 people have died from COVID-19. Currently, the COVID-19 pandemic is still a global challenge as there are continuous genetic variations of the SARS-CoV-2 genome and mutations in the S protein are increasingly reported (2). A new variant of SARS-CoV-2 (Omicron) has more than 50 mutations and is spreading rapidly with an average doubling time of 2 days, and has taken over globally including dividing into subvariants with even more diversity and transmissibility (3). Vaccination may be the most efficient strategy, which is crucial for controlling the COVID-19 pandemic. According to data released by the Chinese National Health Commission on March 19, 2022, more than 3.22 billion COVID-19 vaccine doses have been administered on the Chinese mainland (http://www.nhc.gov.cn/xcs/yqjzqk/list_gzbd.shtml), and nearly 1.24 billion people have been fully vaccinated.

Rheumatoid arthritis (RA) is a chronic autoimmune and inflammatory disorder, which occurs when your immune system attacks your own body's tissues by mistake. RA typically affects the joints, but the systemic inflammatory process can also cause damages on a wide variety of body systems, including the skin, eyes, lungs, heart, and blood vessels (4). Previous studies have shown that he immunogenicity of the pneumococcus vaccine is reduced in RA patients treated with methotrexate (5). Hepatitis B virus vaccines may be less immunogenic in RA patients receiving anti-TNF therapy (6). A study has found impaired antibody responses to the BNT162b2 messenger RNA coronavirus disease 2019 vaccine in RA patients (7). Certain therapies (anti-TNF, anti-IL17, anti-IL6, anti-IL12/23) did not appear to affect seroconversion rates, whereas anti-CD20 and anti-CTLA-4 resulted in poorer responses in rheumatic and non-rheumatic patients treated with immunosuppressive agents (7, 8). The COVID-19 pandemic created concerns about immunosuppression in autoimmunity (9). Although vaccination is recommended for RA patients, they are still anxious about getting vaccinated as the patients with autoimmune disease showed an attenuated humoral response to SARS-CoV-2 vaccination (10), and research showed that immunosuppressed status is associated with an increased risk of COVID-19 infection despite vaccination (11). However, these findings are mostly based on mRNA vaccines (12). It is still unclear whether disease-modifying anti-rheumatic drug (DMARD) treatment can affect inactivated SARS-CoV-2 vaccine-induced seropositivity, which are main types of vaccines used in China.

In this study, we evaluated the safety and immunogenicity of inactivated SARS-CoV-2 vaccine in RA patients and provided further evidence for RA patients to receive inactivated SARS-CoV-2 vaccines.

## Materials and Methods

### Study Design

**Figure 1:**
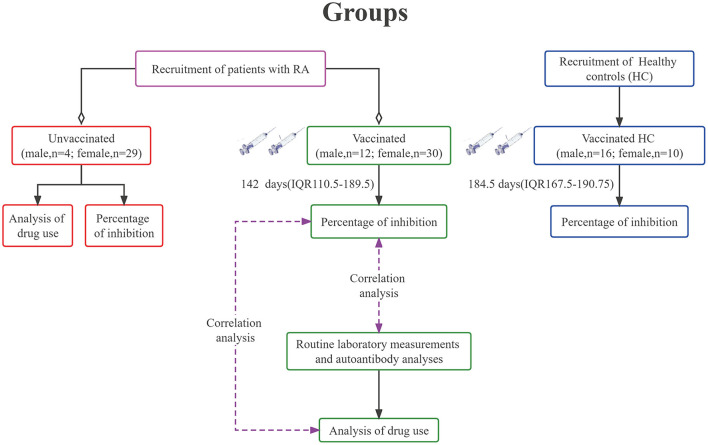
A standard flow diagram for this study.

RA patients and healthy controls received two doses of vaccine mainly between May and August 2021. Blood samples were collected around December 2021. The study population consisted of 75 RA patients and 26 healthy controls (HC), the baseline characteristics of whom were shown in Table 1. Forty-two of 75 patients (56%) received two doses of inactivated SARS-CoV-2 vaccines, and all the HC group were injected twice with the same dose of inactivated SARS-CoV-2 vaccines (Sinovac Life Sciences, Beijing, China, 3μg/0.5mL). Each participant was injected with 0.5 mL of vaccine each time. A standard flow diagram for this study has been shown in Figure 1.

**Table 1:** Baseline characteristics of unvaccinated and vaccinated RA patients, as well as vaccinated healthy controls.

	**RA patients (n = 75)**		
	**Unvaccinated (*n =* 33)**	**Vaccinated (*n =* 42)**	***p*** **-value**
Age, yrs, median (IQR)	62 (45–69)	56 (45.5–60)	0.070
Female sex, *n* (%)	29 (87.88)	30 (71.4)	0.084
**RA disease characteristics**			
AKA positivity, *n* (%)	20 (62.5)	27 (77.14)	0.191
RA disease duration, yrs, median (IQR)	6 (3–15)	8.5 (5.02–17.79)	0.002
RF-IgA, U/ml, median (IQR)	29.69 (1.30–189.47)	41.4 (7.06–154.8)	0.476
RF-IgG, AU/ml, median (IQR)	86.25 (12.70–231.25)	144 (70.2-388)	0.278
RF-IgM, AU/ml, median (IQR)	113.35 (16.13–383.50)	299 (125.25–643.5)	0.170
Anti-CCP antibody, U/ml, median (IQR)	223 (34.13–630.50)	419 (118–770)	0.459
ESR, mm/h, mean (SD)	49.97 (27.78)	46.2 (29.5)	0.761
C-RP, mg/l, median (IQR)	16.04 (7.05–55.26)	7.45 (1.7–37.8)	0.155
NEUT%, mean (SD)	67.50 (20.02)	61.4 (15.7)	0.208
LYMPH%, median (IQR)	20.30 (13.6–34)	24.31 (13.60–33.3)	0.705
**DMARD therapy**			
csDMARDs (Monotherapy), *n* (%)	10 (34.48)	21 (50)	0.195
bDMARDs, *n* (%)	7 (24.14)	9 (21.43)	0.788
bDMARDs (Monotherapy), *n* (%)	0 (0)	1 (11.11)	1.000
JAK inhibitors, *n* (%)	8 (27.59)	1 (2.38)	0.006
Prednisone, *n* (%)	5 (17.24)	6 (14.29)	0.996
TGT, *n* (%)	9 (31.03)	6 (14.29)	0.089
	**RA patients (*n* = 42)**	**Healthy controls (*n* = 26)**	
Age yrs, median (IQR)	56 (45.5–60)	44.5 (36.25–53)	0.001
Female sex, *n* (%)	30 (71.4)	10 (42.3)	0.007
Mean interval between 2nd vaccination and sampling, days, median (IQR)	142 (110.5–189.5)	184.5 (167.5–190.75)	0.003

*AKA, anti-keratin antibody; RF, rheumatoid factor; CCP, cyclic citrullinated peptide; ESR, erythrocyte sedimentation rate; C-RP, c-reactive protein; NEUT, neutrophils; LYMPH, lymphocytes; DMARD, disease modifying antirheumatic drugs; cs, conventional synthetic; b, biologic; JAK, Janus kinase; TGT, tripterygium glycosides tablet*.

The inclusion criteria met the 1987 American College of Rheumatology (ACR) criteria. Patients with moderate to high activity (Disease Activity Score in 28 Joints (DAS28) > 3.2) were aged between 18 and 70 years, and there was no restriction on gender and disease course. Those who voluntarily take the test have signed the informed consent. The exclusion criteria were those who did not meet the inclusion criteria or those with overlapping rheumatic diseases such as systemic lupus erythematosus, sjogren's syndrome, severe osteoarthritis, or those with mental or legal disabilities, and those who were unwilling to sign informed consent. The key exclusion criteria included no history of exposure to COVID-19 and positive PCR tests for SARS-CoV-2. The HC were healthy volunteers who did not receive immunosuppressive treatments. The study was approved by Yunnan Provincial Traditional Medicine Hospital Ethics Committee (NO. YNSZLL-AF-027-2020/07), and registered at ClinicalTrials.gov (NCT05191368). Written consent was obtained from all participants before sampling.

### Routine Laboratory Measurements and Autoantibody Analyses

Blood samples were collected from RA patients as part of routine clinical testing. In this study, AKA (anti-keratin antibody) positivity was detected using indirect immunofluorescence (13). Rheumatoid factor (RF)-IgA was detected by ELISA (13). RF-IgG, RF-IgM, and anti-CCP antibodies were measured by chemiluminescence (14, 15). ESR (erythrocyte sedimentation rate) was detected by ESR analyzer (15). C-RP (c-reactive protein) was detected by immunoturbidimetry (16). NEUT% (percentage of neutrophils) and LYMPH% (percentage of percentage) were calculated by a calculation method (17).

### Percent Inhibition Measurement

Percent inhibition measurement was based on the competitive inhibition of the interaction between the SARS-CoV-2 spike protein and the angiotensin-converting enzyme 2 cell surface receptor (18). Serum samples were collected and the SARS-CoV-2 S protein Neutralizing Antibody Test ELISA kit (SN: EKnCo v001, Frdbio, Wuhan, China) was used to measure the percentage of inhibition, which was calculated using the following formula: percentage of inhibition = (1–(OD 450 of sample/OD 450 of negative control) × 100%. Percentage of inhibition ≧20% means anti-SARS-CoV-2 neutralizing antibodies (NAbs) detected (seropositive), and percentage of inhibition < 20% means anti-SARS-CoV-2 NAbs not detected (seronegative) (19). The criterion for the validity of experimental data is: OD450 (or OD450/OD630) of negative control wells > 0.900, and OD450 (or OD450/OD630) of positive control wells < 0.300. The experiment was carried out in strict accordance with the instructions. A Variskan flash automatic microplate reader (Thermo Scientific, USA) was used to measure absorbance at 450 nm.

### Statistical Analysis

The SPSS 22.0 software was used to perform the statistical analysis. Shapiro–Wilk test was used to analyze whether the data followed a normal distribution. If the data were normally distributed, they were presented as mean ± SD (standard deviation), and two independent samples *t-*tests were used. If they were not normally distributed, they were presented as median (IQR, interquartile range), and Kolmogorov-Smirnov test was adopted. The enumeration data were described by frequency and percentage, and χ^2^ test was used. *p* < 0.05 was considered to be statistically significant.

## Results

### Participant's Characteristics

As shown in Table 1, the age of unvaccinated and vaccinated RA patients was respectively 62 (45–69) years and 56 (45.5–60) years, and no significant difference was observed (*p* = 0.070). Among unvaccinated RA patients, 4 (12.12%) were male and 29 (87.88%) were female, while the percent of male and female was 28.57% and 71.43% among vaccinated RA patients, and no significant difference was evident (*p* = 0.084). The disease characteristics of unvaccinated RA patients were not significantly different from those of vaccinated RA patients (Table 1, *p*-values > 0.05) other than the duration of disease (*p* = 0.002) with longer disease duration in vaccinated RA patients [8.5 (5.02–17.79) years]. All RA patients have received continuous therapy with conventional synthetic (cs), biological (b), or targeted synthetic (ts) DMARDs, as well as Janus kinase (JAK) inhibitors, prednisone or tripterygium glycosides tablet (TGA). csDMARDs was used in 31 (43.66%) patients, and 17 (22.54%) patients were treated with bDMARDs. In total 9 (12.68%) patients were given JAK inhibitors, and 11 (15.49%) patients were taking prednisone. In total 15 (21.13%) patients were prescribed with TGA. There was a significant difference in the proportion of patients using JAK inhibitors between unvaccinated (27.59%) and vaccinated (2.38%) in RA patients (Table 1, *p* = 0.006). While, the other treatment strategies were similar between unvaccinated and vaccinated RA patients (Table 1, *p*-values > 0.05).

The HC group (44.5 (36.25–53) years) were younger (Table 1, *p* = 0.001) than RA patients (56 (45.5–60) years), and there was also a significant difference in the sex ratio between the two groups (*p* = 0.007). The interval time between the second vaccination and serum sampling was significantly shorter in RA patients than HC (RA vs. HC: 142 (110.5–189.5) days vs. 184.5 (167.5–190.75) days, *p* = 0.003).

### Vaccine Safety

It is relatively safe for RA patients to receive two doses of inactivated SARS-CoV-2 vaccines. Side effects and adverse reactions were comparable to the HC group. No serious adverse events were reported among HC. In total 2 (4.44%) of the RA patients developed significant joint pains, knee effusion, and swelling of the hands and feet after the second dose. However, it is still unclear whether the adverse events were related to the vaccination.

### Percentage of Inhibition

**Figure 2:**
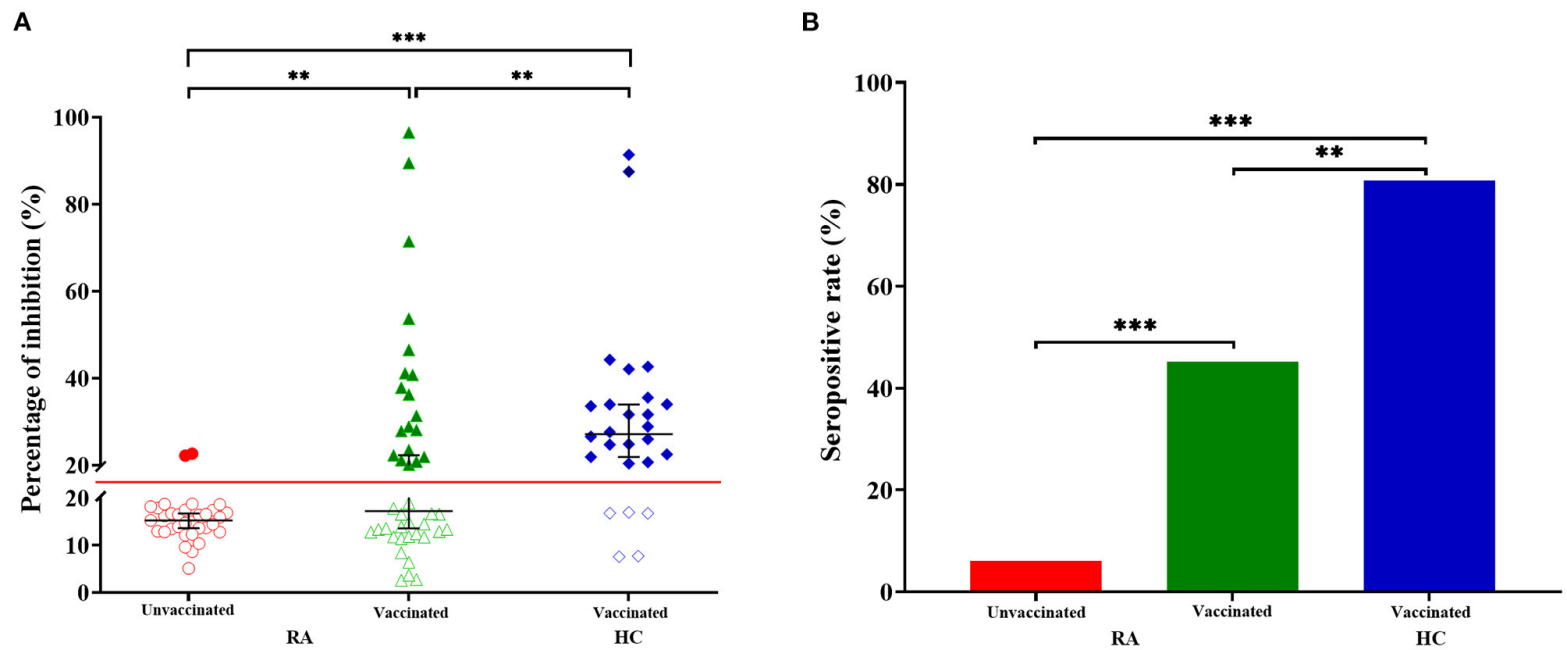
Anti-SARS-CoV-2 antibody responses from RA patients and HC. **(A)** The percentage of inhibition from individuals who have not been vaccinated RA patients (*n* = 33), vaccinated RA patients (*n* = 42) and healthy controls (HC, *n* = 26). Symbols show individual values, red line show the cut-off value (20%), and black horizontal bars show medians. Statistical analysis was done using the Kolmogorov-Smirnov test. Data were presented as median (IQR). **p < 0.01; ***p < 0.001. **(B)** Seropositive rate of unvaccinated RA patients (*n* = 33), vaccinated RA patients (*n* = 42) and healthy controls (HC, *n* = 26). Statistical analysis was done using the χ^2^ test. **p < 0.01; ***p < 0.001.

Compared with the HC group, the percentage of inhibition was significantly lower in RA patients receiving two doses of vaccines (Figure 2A, RA: median 17.24%, IQR 12.68–29.50; HC: median 27.08%, IQR 20.58–34.36; *p* = 0.006). While, the percentage of inhibition was the lowest in RA patients who have not been vaccinated (Figure 2A, median 15.26%, IQR 12.89–17.43), which was significantly lower than vaccinated RA patients (Figure 2A, *p* = 0.003) or HC (Figure 2A, *p* = 1.782 × 10^−7^). 18 (80.77%) of 26 HC had a higher seropositivity, compared with 19 (45.24%) of 42 vaccinated RA patients (Figure 2B, *p* = 0.004) and 2 (6.06%) of 33 RA patients who had not received the vaccination (Figure 2B, *p* = 5.173 × 10^−9^). Furthermore, the difference was also significant between vaccinated and unvaccinated RA patients (Figure 2B, *p* = 0.0002).

### Inhibition Percent in Relation to Levels of Laboratory Indicators

**Figure 3:**
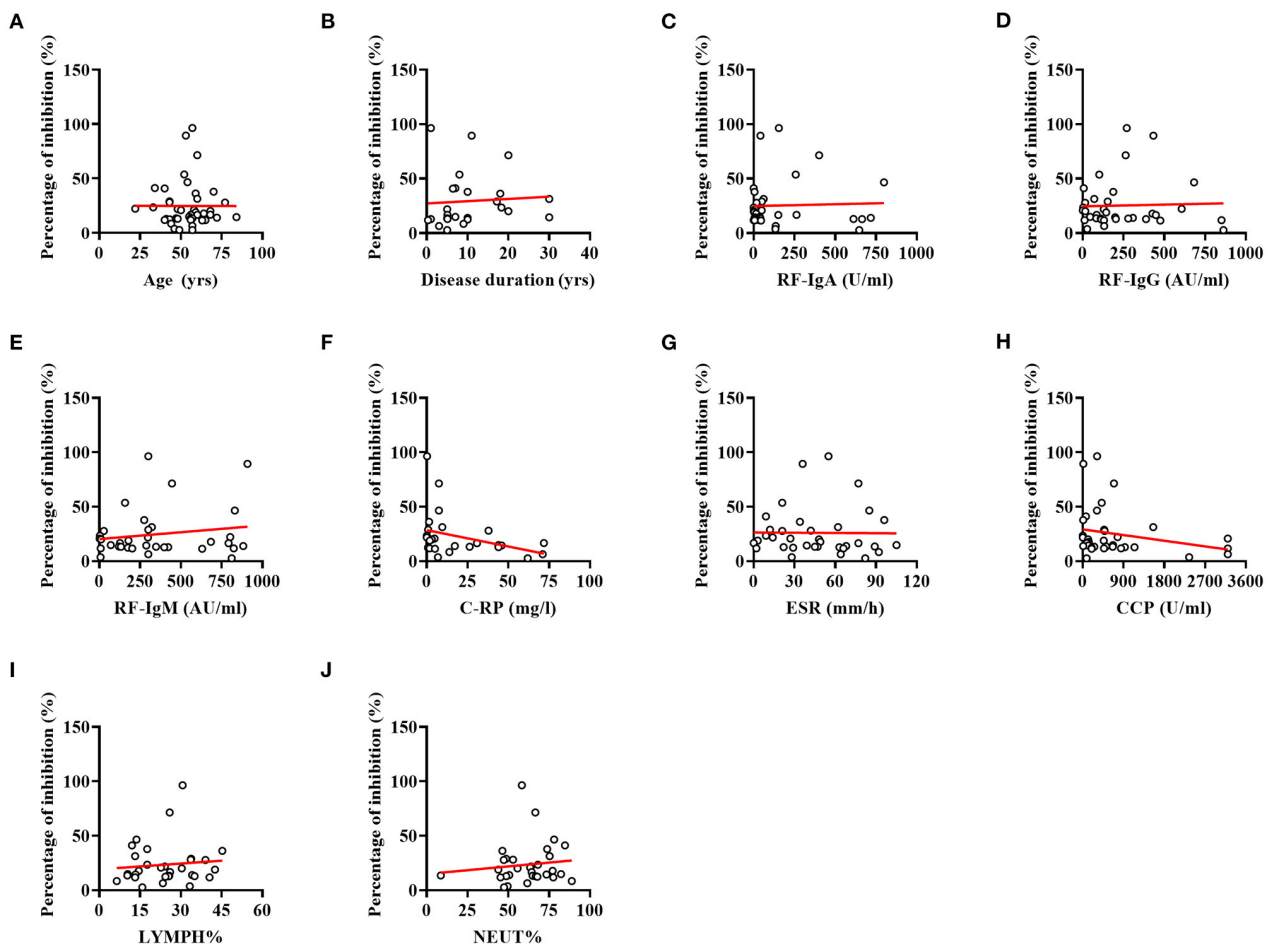
Correlations between indices for RA and percentage of inhibition. **(A)** Correlation between the age of patients and percentage of inhibition. **(B)** Correlation between the disease duration and percentage of inhibition. **(C)** Correlation between the RF-IgA and percentage of inhibition. **(D)** Correlation between the RF-IgG and percentage of inhibition. **(E)** Correlation between the RF-IgM and percentage of inhibition. **(F)** Correlation between the C-RP and percentage of inhibition. **(G)** Correlation between the ESR and percentage of inhibition. **(H)** Correlation between the Anti-CCP antibody and percentage of inhibition. **(I)** Correlation between the LYMPH% and percentage of inhibition. **(J)** Correlation between the NEUT% and percentage of inhibition. No significant correlations were evident between indices for RA and percentage of inhibition (all *p*-values > 0.05).

Spearman correlation analysis revealed that antibody responses to SARS-CoV-2 did not differ between RA patients according to their age (Figure 3A, r = 0.004, *p* = 0.980) and disease duration (Figure 3B, r = 0.363, *p* = 0.075). Furthermore, lower NAbs percentage of inhibition was not caused by RA-induced immune impairments, including immune-related indicators: RF (Figure 3C, IgA: r = 0.017, *p* = 0.925; Figure 3D, IgG: r = −0.108, *p* = 0.535; and Figure 3E, IgM: r = 0.041, *p* = 0.819), C-RP (Figure 3F, C-reactive protein: r = −0.361, *p* = 0.064), ESR (Figure 3G, erythrocyte sedimentation rate: r = −0.146, *p* = 0.401), and anti-CCP antibody (Figure 3H, anti-cyclic citrullinated peptide antibody: r = −0.286, *p* = 0.095), as well as LYMPH% (Figure 3I, r = 0.093, *p* = 0.611) and NEUT% (Figure 3J, r = 0.121, *p* = 0.509).

## Discussion

In response to the SARS-CoV-2 outbreak and the resulting COVID-19 pandemic, a global competition to develop anti-COVID-19 vaccine has ensued (20). As the vaccination programs progress worldwide, the requirement of credible SARS-CoV-2 antibody assays (to demonstrate a successful vaccine response) will probably remain over the next couple of years (21). Previous studies have provided sufficient data on the safety and immunogenicity of vaccines in healthy individuals, with satisfactory results. Moreover, considering increased risks of COVID-19 infection and severe outcomes, the immune response of RA patients to vaccines needs to be further assessed (22). Effectiveness of mRNA COVID-19 vaccine in autoimmune disease patients have been studied (11, 23). Recent studies have demonstrated an attenuated humoral response to mRNA SARS-CoV-2 vaccination in patients with autoimmune disease (10, 24). However, data on immune response responses to inactivated SARS-CoV-2 vaccine in the RA population remains unknown. Based on the global prevalence of SARS-CoV-2 infection, there should be comprehensive data on immune responses to inactivated vaccines in RA patients. Besides, studies have shown that NAbs level is a good biomarker for the correlate of protection against COVID-19 (25, 26). Thus, we sought to describe the NAbs level in patients with RA who received two injections of inactivated SARS-CoV-2 vaccine.

Inactivated virus vaccines could be prepared through the employment of established physical and chemical methods such as UV light, formaldehyde, and β-propiolactone (27), which have both advantages and disadvantages. It is easy to prepare, safe, and has high-titer NAbs, but it is unsuitable for highly immunosuppressed individuals (28). A study has demonstrated that the seroconversion rates of neutralizing antibodies were 3.3% (2 out of 60) and 95% (57 out of 60) for individuals who had received 2 and 3 doses of vaccine, respectively (29). To our knowledge, this is the first study to investigate the effects of inactivated SARS-CoV-2 vaccine on RA patients. Our data demonstrated that the response of inactivated vaccine-induced immune responses differ between RA patients and HC, consistent with the finding from mRNA vaccines (12). This suggests that RA patients should receive a third or fourth booster dose to mount a comparable antibody response, especially for the rapidly spreading omicron variant (30). A booster dose has proven to be highly effective against COVID-19 and related severe disease and death. The data from Centers for Disease Control and Prevention (CDC) has shown that unvaccinated adults are nearly six times more likely to test positive for COVID-19 and 14 times more likely to die from the virus compared to vaccinated individuals. Therefore, the CDC has updated its guidance to indicate that immunocompromised people can get a fourth COVID-19 shot. In addition, our study showed that the whole course (2 doses) vaccination of inactivated vaccines are safe for RA patients. Adverse symptoms were less severe and mostly transient, and pain at the injection site was the most reported symptom. Regarding abnormal laboratory indicators, two patients developed joint pain in the limbs, knee joint effusion, swelling, and pain in both hands and feet after the second dose of injection, but it is undetermined whether the adverse events are related to the vaccination. Overall, the safety profile for most RA patients was equally good.

RA patients have been taking immunosuppressive or immunomodulatory drugs. Such treatments, e.g., glucocorticoids or DMARDs, may potentially result in decreased responses to vaccination, including establishing and maximizing the immune response (31). Attenuated levels of anti-spike IgG antibodies and neutralization capacity, as well as B cell responsiveness, were reported in RA patients treated with JAK inhibitors (32). JAK inhibitors can also impair IFN-mediated antiviral responses and increase the risk of secondary infection (33). Thus, a temporary suspension of tsDMARDs was recommended to RA patients infected with SARS-CoV-2. Our results showed that the use of JAK inhibitors was significantly different between vaccinated and unvaccinated RA patients, and other indicators were not significantly different (Table 1). The reason why some patients did not receive vaccination in our study maybe that they were taking JAK inhibitors, which may weaken antiviral responses and increase the risk of secondary infection. None of RA patients or HC reported symptoms suggestive of COVID-19 throughout the observation period, and none had a positive SARS-CoV-2 antigen or RT-PCR test. Of note, percentages of inhibition of two RA patients after the second vaccination were higher than 80%, which was 96.50 and 89.50%, respectively. The first patient received a combination drug therapy, including methotrexate, adamu, folic acid, and leflunomide. The second patient received treatments of methotrexate, leflunomide, prednisone, and TGA. These results suggested that csDMARDs and bDMARDs may not affect vaccine-induced antibody responses. It should be noticed that one patient using JAK inhibitors had a low percentage of inhibition (6.40%), which received a second dose of inactivated vaccine on November 1, 2021, and his serum was sampled on December 2. Although the time interval was short, NAbs were negative. It is suggested that JAK inhibitors may inhibit the production of NAbs, which is consistent with other studies (32). However, it is an individual case, and the relationship between JAK inhibitors and NAb inhibition rate is unclear, and further research is needed to verify. Conflicting results were reported in patients using methotrexate (8, 34). We did not find that methotrexate hampers humoral response to vaccine in our case series. Herein, twenty-one patients (7 males, 14 females) were treated with csDMARDs alone, all males were negative. It is suggested that the vaccine effect may be lower in males compared with females, and the correlation between gender and NAb inhibition rate needs to be further explored. In addition, six RA patients used prednisone, 2 of whom were positive for NAbs. Because some patients used drugs such as csDMARDs and Tripterygium glycosides tablet simultaneously, the relationship between drug use and inhibition rate was complicated, and the sample size needs to be expanded to further research. See the Table 1 for more details.

In unvaccinated RA patients, two patients showed >20% inhibition. It may be that some people may have been directly or indirectly exposed to other similar coronaviruses in their lives, which induced the production of antibodies in their bodies. Studies have shown that late humoral and cellular responses are detected in some people who have not been exposed to SARS-CoV-2 (35). Furthermore, when qualitative experimental methods are evaluated, the clinical diagnosis is unknown in many cases. Therefore, it is understandable that the antibody inhibition rate was slightly higher than 20% in the serum of two individual patients in this study. Of course, it is undeniable that this is also related to individual differences.

One limitation of our study was the age and sex mismatch between the RA group and the HC group, but fortunately, age was not the major influencing factor (36). Studies have found that attenuated anti-SARS-CoV-2 antibody response to vaccination in patients with rheumatic diseases, and non-responders and responders were equally distributed across all age categories (37). In addition, there was no significant correlation between age and the inhibition rate of NAb produced by inactivated vaccine (19), which was consistent with the results of this study. Whether there is a correlation between age and NAb inhibition rate produced by inactivated vaccine needs to be further verified by expanding the sample size. In addition, previous studies have shown that males had lower serological response to SARS-CoV-2 vaccination (38). Male gender had been implicated in the poor vaccination responsiveness in general population (39–41). In this study, with the same injection of 2 vaccines, there were more males in the HC (16 males, 10 females), and fewer males in the RA group (12 males, 30 females), while the NAb inhibition rate in the RA group was still lower. These all suggested that RA patients exhibited lower humoral responses to vaccines than the HC group. Another limitation was that the time interval between the second vaccination and serum sampling was significantly different between RA patients and HC, shorter in RA patients than in the HC group. There is evidence showing declines in vaccine effectiveness against COVID-19 with increasing time (36), and a significant antibody decline was observed at 3-month post-vaccination (42). In this study, the percentage of inhibition to SARS-CoV-2 was still lower in RA patients with a relatively shorter interval. This may be related to the use of immunosuppressants in RA patients, which needs to be confirmed by further studies.

In summary, our study suggests that 2-dose of inactivated vaccine-induced SARS-COV-2 antibody responses differ in RA patients and healthy subjects, emphasizing the importance of a third or fourth vaccination in RA patients. Future studies with larger sample sizes and longer follow-up time are required to define the optimal vaccine strategy and clarify whether DMARDs should be suspended in RA patients to increase the positive responses to vaccination and better protect this vulnerable population.

## Data Availability Statement

The original contributions presented in the study are included in the article/supplementary material, further inquiries can be directed to the corresponding author/s.

## Ethics Statement

The studies involving human participants were reviewed and approved by Yunnan Provincial Traditional Medicine Hospital Ethics Committee (NO. YNSZLL-AF-027-2020/07). The patients/participants provided their written informed consent to participate in this study.

## Author Contributions

All authors listed have made a substantial, direct, and intellectual contribution to the work and approved it for publication.

## Funding

This work was supported by the National Natural Science Foundation of China (31960178, 82160923, 81960863, and 81960870); Construction Project of National Traditional Chinese Medicine Clinical Research Base (2018 No. 131); Yunnan Provincial Fund for Medical Research Center: Clinical Evaluation and Basic Research on the Treatment of rheumatoid arthritis and gout by Traditional Chinese medicine (202102AA310006); Clinical Trial for the Treatment of Rheumatoid Arthritis with Warming yang and Smoothening Meridians (201507001-07, Registration Number: ChiCTR-INR-16010290); Clinical Cooperative Project of Chinese and Western Medicine for Major and Knotty Diseases; Yunnan Provincial Key Laboratory Construction Project Funding; Yunnan Provincial Key Laboratory of Chinese Medicine Rheumatology and Immunology; Yunnan Provincial Ten Thousands Program Famous Doctor Special; Yunnan Province Qingguo Wang Expert Workstation Construction Project (202005AF150017); Yunnan Applied Basic Research Projects-Union Foundation [2019FF002(-031)]; Applied Basic Research Programs of Science and Technology Commission Foundation of Yunnan Province (2019FA007); Scientific Research Fund Project of Yunnan Provincial Department of Education (2021Y461).

## Conflict of Interest

The authors declare that the research was conducted in the absence of any commercial or financial relationships that could be construed as a potential conflict of interest.

## Publisher's Note

All claims expressed in this article are solely those of the authors and do not necessarily represent those of their affiliated organizations, or those of the publisher, the editors and the reviewers. Any product that may be evaluated in this article, or claim that may be made by its manufacturer, is not guaranteed or endorsed by the publisher.
